# The MAPK/ERK Pathway and the Role of DUSP1 in JCPyV Infection of Primary Astrocytes

**DOI:** 10.3390/v13091834

**Published:** 2021-09-14

**Authors:** Michael P. Wilczek, Francesca J. Armstrong, Remi P. Geohegan, Colleen L. Mayberry, Jeanne K. DuShane, Benjamin L. King, Melissa S. Maginnis

**Affiliations:** 1 Department of Molecular and Biomedical Sciences, University of Maine, Orono, ME 04469, USA; michael.wilczek@maine.edu (M.P.W.); francesca.armstrong@maine.edu (F.J.A.); remi.geohegan@maine.edu (R.P.G.); colleen.mayberry@maine.edu (C.L.M.); jeanne.dushane@maine.edu (J.K.D.); benjamin.king@maine.edu (B.L.K.); 2 Graduate School in Biomedical Sciences and Engineering, University of Maine, Orono, ME 04469, USA

**Keywords:** JC polyomavirus, progressive multifocal leukoencephalopathy (PML), astrocytes, RNA sequencing, bioinformatics

## Abstract

JC polyomavirus (JCPyV) is a neuroinvasive pathogen causing a fatal, demyelinating disease of the central nervous system (CNS) known as progressive multifocal leukoencephalopathy (PML). Within the CNS, JCPyV predominately targets two cell types: oligodendrocytes and astrocytes. The underlying mechanisms of astrocytic infection are poorly understood, yet recent findings suggest critical differences in JCPyV infection of primary astrocytes compared to a widely studied immortalized cell model. RNA sequencing was performed in primary normal human astrocytes (NHAs) to analyze the transcriptomic profile that emerges during JCPyV infection. Through a comparative analysis, it was validated that JCPyV requires the mitogen-activated protein kinase, extracellular signal-regulated kinase (MAPK/ERK) pathway, and additionally requires the expression of dual-specificity phosphatases (DUSPs). Specifically, the expression of DUSP1 is needed to establish a successful infection in NHAs, yet this was not observed in an immortalized cell model of JCPyV infection. Additional analyses demonstrated immune activation uniquely observed in NHAs. These results support the hypothesis that DUSPs within the MAPK/ERK pathway impact viral infection and influence potential downstream targets and cellular pathways. Collectively, this research implicates DUSP1 in JCPyV infection of primary human astrocytes, and most importantly, further resolves the signaling events that lead to successful JCPyV infection in the CNS.

## 1. Introduction

JC polyomavirus (JCPyV) is a human-specific virus that infects astrocytes and oligodendrocytes, leading to the fatal, demyelinating disease, progressive multifocal leukoencephalopathy (PML) [1,2,3,4,5]. JCPyV infects most of the population, causing an asymptomatic persistent infection in the kidneys of healthy individuals [6,7,8,9,10]. However, during immunosuppressive events, the virus can become reactivated in the central nervous system (CNS), infecting glial cell types [9,11,12]. If left untreated, PML can be fatal within a few months [13]. Individuals most at risk for this disease include those undergoing immunomodulatory therapies, such as natalizumab for multiple sclerosis (MS) or people who are HIV positive [1,14,15,16]. While there have been advancements in treatment, which address the underlying immunosuppression aligned with supportive care, resulting in improved survival rates, a sufficient cure for the disease is still lacking [16,17,18,19]. Beyond these therapies, research has demonstrated that JCPyV encodes microRNAs (miRNAs) [20]. Specifically, miR-J1-5p, a mature miRNA produced in the JCPyV infectious cycle and expressed in PML tissue, may have a repressive role in viral replication [21,22]. Thus, continuing to uncover the molecular basis of JCPyV infection can potentially uncover new therapeutic approaches for disease treatment and diagnostic tools for PML [21,22].

Limited approaches to studying JCPyV have resulted in a reliance on in vitro cell culture models for advancements in the mechanistic understanding of JCPyV infection [23,24,25,26,27,28]. Specifically, the most widely used cell model of JCPyV infection is SVG-A cells (referred herein as SVGAs), immortalized human fetal glial cells, mostly composed of astrocytes, that constitutively express SV40 Large T antigen (T Ag) to increase viral replication [29,30,31]. Our laboratory recently characterized JCPyV infection in primary normal human astrocytes (NHAs) as a tool to understand the infectious cycle [32,33]. When JCPyV infection in NHAs was compared to SVGAs, it was discovered that JCPyV infection was delayed in primary astrocytes, mostly due to the inadequate production of JCPyV Large T Ag compared to levels of this viral protein in cells immortalized with SV40 Large T Ag [32]. This research corroborated the observations of Kondo et. al. in a chimeric mouse model; astrocytes were readily infected, but expressed low levels of the late viral gene product VP1 [5,33,34,35]. Further, we demonstrated that the immortalization of NHAs with SV40 Large T Ag, termed NHA-Ts, restored levels of VP1 expression comparable to JCPyV infection in SVGAs [32]. These results illustrate that expression of SV40 T Ag greatly contributed to the delayed progression in JCPyV infection observed in NHAs when compared to SVGAs [32]. However, expression of SV40 Large T Ag to immortalize cells and enhance JCPyV replication may also dysregulate the cell cycle and activate cellular signaling pathways, confounding results in immortalized cell types [36,37].

Viral reprogramming of cells is complex and challenging to assess through targeted approaches and may be further complicated by cell-type dependent differences. To date, only a few studies report a complete cellular transcriptomic profile of cells infected with JCPyV [34,38,39]. In two earlier studies, a microarray analysis was performed in primary human astrocytes [38] and in cells differentiating from human multipotential human CNS progenitor cells (NPCs) into progenitor-derived astrocytes (PDAs) [34]. The study in primary human astrocytes revealed that many transcripts upregulated upon JCPyV infection encoded proteins involving cellular proliferation, signaling pathways, such as the transforming growth factor β (TGF-β) receptor 1, and other regulatory events, such as inflammatory responses [38]. They validated the gene expression data by specifically examining genes involved in the cell cycle or cellular proliferation, demonstrating the upregulation of these proteins at 5 or 15 days post-infection by western blot [38]. While these earlier studies established a foundation for understanding gene expression changes in JCPyV-infected cells, the majority of JCPyV studies have been performed in SVGAs.

Previous research established that JCPyV uses the cellular, mitogen-activated protein kinase, extracellular signal-regulated kinase (MAPK/ERK) pathway to reprogram the cell and support viral infection [40,41]. The MAPK/ERK pathway is important in determining cell fate in proliferation, differentiation, and survival [42]. It was determined that both SVGAs and a primary kidney cell line, renal proximal tubule epithelial cells (RPTECs) required ERK phosphorylation for infection as U0126, an inhibitor of the kinase MEK, upstream of ERK, reduced infection in both cell types [40]. It was further validated that kinases in this pathway, BRAF, CRAF, MEK1, and MEK2, were all critical for infection in SVGAs [43].

Other viruses activate the MAPK/ERK pathway to reprogram cells and support viral infection [40,44,45,46]. However, there has been little research to understand how the negative regulatory mechanisms of the MAPK/ERK pathway may play in viral infection, including the function of dual specificity phosphatases (DUSPs). DUSPs are made up of a family of protein tyrosine phosphatases that strongly regulate and deactivate MAPK signaling [47,48,49,50]. Typical DUSPs, or phosphatases that contain a MAP kinase-binding (MKB) motif or the kinase-interacting motif (KIM), are dynamic proteins further classified by their subcellular cytoplasmic and/or nuclear localization [51,52]. One of the most well-studied DUSPs in glial cells is DUSP1 [47]. This phosphatase is found in the nucleus and binds to JNK, p38, and ERK1/2, leading to the dephosphorylation and inactivation of these kinases [50]. Interestingly, cellular levels of ERK can be sustained by targeting DUSP1 for degradation via the ubiquitin/proteasome pathway [53,54,55], further highlighting the complexity of the regulatory processes of the MAPK/ERK pathway. DUSPs, specifically DUSP1, have been implicated in other viral infections as well, including hepatitis C virus (HCV), vaccinia virus (VACV), human respiratory syncytial virus (RSV), and Sendai virus (SeV) [56,57,58]. Through ERK phosphorylation, DUSP1 is upregulated and stabilized, further promoting viral replication and release during VACV infection [56]. Moreover, DUSP1 may also be differentially expressed during coronavirus infection [59], highlighting the importance of this protein during infection across many viral families. Conversely, DUSP1 is also known to be involved in the inflammatory response [60], as research has demonstrated that knockdown of the protein can reduce HCV infection, as it promotes the induction of interferon stimulated genes (ISGs) [57]. Altogether, research has demonstrated the role of DUSP1 in the MAPK/ERK pathway and the influence it has on alternative pathways can either enhance or reduce viral infection.

The purpose of this study was to elucidate the cellular pathways, with a particular focus on the MAPK/ERK pathway and the regulatory mechanisms involved during JCPyV infection of primary astrocytes. This was established through a comparative approach, using RNA sequencing (RNA-seq), bioinformatics analysis, and complementary cell-based assays to define the host transcriptome profile in primary astrocytes during JCPyV infection. These data further validate the importance of ERK1/2 in JCPyV infection, reveal novel regulatory roles of the MAPK/ERK pathway, and highlight the importance of DUSP1 expression during JCPyV infection in primary astrocytes.

## 2. Materials and Methods

### 2.1. Cells and Viruses

The maintenance and description of normal human astrocytes (NHAs) (passage 1 [P1]) were previously reported [32]. Briefly, NHAs were cultured in astrocyte growth medium and supplemented with SingleQuots supplements and 1% penicillin–streptomycin (P–S). The donor was a 19-week-gestation female with no detected levels of HIV, hepatitis B virus (HBV), or hepatitis C virus (HCV). All experiments were performed at low passages (P2 to P10). SVGAs were generously provided by the Atwood Laboratory (Brown University, Providence, RI, USA) and cultured in complete minimum essential medium (MEM) (Corning, Corning, NY, USA), with 10% fetal bovine serum (FBS), 1% P–S and 0.1% Plasmocin prophylactic (InvivoGen, San Diego, CA, USA). The generation and maintenance of NHA-Ts are previously described [32]. All cell types were grown in a humidified incubator at 37 °C with 5% CO_2_.

The generation and production of either lysate or purified JCPyV strains of Mad-1/SVEΔ were described previously [61,62], and the strain was kindly provided by the Atwood laboratory (Brown University).

### 2.2. siRNA Treatment

NHAs and NHA-Ts were plated for ~70% confluency, while SVGAs were plated to ~50% confluency in 96 well plates and transfected with siRNAs specific for ERK1/2, epidermal growth factor receptor (EGFR) control (Cell Signaling Technology, Danvers, MA, USA), or DUSP1 (Santa Cruz Biotechnology, Dallas, TX, USA). The EGFR control was used as a non-specific siRNA in parallel with either ERK1/2 or DUSP1 siRNA. A final concentration of 1 pmol of siRNA/well of ERK1/2, and a final concentration of 4 pmol of DUSP1 siRNA/well was used with RNAiMax transfection reagent (Invitrogen, Waltham, MA, USA) by mixing transfection complexes in Opti-MEM, reduced serum medium (Gibco, Waltham, MA, USA) at RT for 7 min. Complexes were added to the cells (10 µL/well) and incubated at 37 °C for 72 h for the ERK1/2 siRNA and 24 h for the DUSP1 siRNA. These timepoints resulted in the greatest level of knockdown for each protein, respectively. Protein reduction was measured by ICW assay [43,63] or cells were infected with JCPyV following treatment of siRNA.

### 2.3. JCPyV Infection

NHAs, SVGAs, and NHA-Ts were seeded into 96-well plates with approximately 10,400 cells/well to achieve ~70% confluency at the time of infection. Cells were infected (multiplicities of infection [MOIs] as indicated in the figure legend) with 42 μL/well of MEM containing 10% FBS, 1% P–S, and 0.1% Plasmocin prophylactic, at 37 °C for 1 h. After the initial 1 h, cells were fed with the respective medium at 100 μL/well and incubated at 37 °C for the duration of the infection. Experiments in which the percent infection was quantified following treatment. Cells were fixed at 48 h and stained by indirect immunofluorescence for the viral protein, T Ag.

### 2.4. Indirect Immunofluorescence Staining for Quantitation of JCPyV Infection

Following infection, all cell types were stained for T Ag at room temperature (RT). NHAs, SVGAs, and NHA-Ts were fixed with 4% paraformaldehyde (PFA) for 10 min and washed with 1× phosphate-buffered saline (PBS) with 0.01% Tween (PBS-T). Cells were permeabilized for 15 min using PBS-0.5% Triton X-100 and then blocked with PBS-T containing 10% goat serum for 45 min. Cells were then stained using antibodies specific for viral proteins at RT for 1 h (Table 1). Following 1° antibody incubation, cells were washed three times in PBS-T and counterstained with a 2° antibody, specifically an anti-mouse Alexa Fluor 594 (Table 1) for 1 h. Cells were subsequently washed with PBS-T and counterstained using DAPI (4′,6-diamidino-2-phenylindole) at RT for 5 min. Cells were washed with PBS-T twice and stored in PBS-T at 4 °C until further analysis.

To visualize the cells expressing the nuclear, viral protein T Ag, a Nikon Eclipse Ti epifluorescence microscope (Micro Video Instruments, Inc., Avon, MA, USA) equipped with a 20× objective was used. Percent infection of each well was quantified by counting the T Ag-positive cells over the total number of DAPI-positive cells in five fields of view/well. The DAPI-positive cells were determined by using a binary algorithm in the Nikon NIS-Elements Basic Research software (version 4.50.00, 64 bit). The algorithm created an accurate number of cells in each field by separating them based on intensity, diameter, and circularity [32,40,43,64,65].

### 2.5. ICW Assay and LI-COR Quantification

Protein expression measuring total or phosphorylated ERK1/2 and total DUSP1 were performed using an ICW assay [43,63]. Following siRNA treatment, cells were fixed in 4% PFA, washed with PBS-T and permeabilized with 1× PBS-0.5% Triton X-100 at RT for 15 min. Cells were blocked with Tris-buffered saline (TBS) Odyssey buffer (LI-COR, Lincoln, NE, USA) at RT for 1 h. Cells were stained with the appropriate antibody (dilutions indicated in Table 1) in TBS Odyssey blocking buffer at 4 °C for ~16 h while rocking. The next day, cells were washed with PBS-T three times and incubated with the corresponding secondary antibody in Table 1 and CellTag (1:500, LI-COR) for 1 h. Cells were washed with PBS-T, aspirated and the bottom of the plate was cleaned with 70% EtOH prior to scanning. Plates were weighted down using a silicone mat (LI-COR) and the lid was removed before imaging, using the LI-COR Odyssey CLx infrared imaging system to detect both the 700- and 800-nm intensities. Plates were read at medium quality, at 42-μm resolution, with a 3.0 mm focus offset. Once scanning was complete, the 700- and 800-nm channels were aligned and ICW analysis was performed in Image Studio software (version 5.2). Quantification was determined by subtracting the background from the 800-nm channel (secondary antibody alone) from each well, in which the protein of interest was being measured. Next, the ratio of the 800-nm channel (protein of interest) signal to the 700-nm channel (CellTag) in each well was determined.

### 2.6. Relative Quantification of DUSP1 Transcript Levels by qPCR

NHAs, SVGAs, and NHA-Ts were plated to 70% confluence in 96-well plates. The medium was removed and cells were infected with JCPyV (MOI = 0.1 FFU/cell (the same virus prep used in the RNA-seq infection)) in 42 μL of the respective cell medium and incubated at 37 °C for 0, 24, 48, and 96 h. RNA was extracted as outlined previously and was converted to cDNA with the iScript cDNA synthesis kit (Bio-Rad) using 1 μg of RNA. Primers for DUSP1 (F: GGATACGAAGCGTTTTCGGC, R: AGAGGTCGTAATGGGGCTCT) [66] and glyceraldehyde-3-phosphate dehydrogenase (GAPDH) were used as housekeeping genes [67]. After creating the master mix of qPCR reagents with the iQ SYBR green supermix (Bio-Rad, Hercules, CA, USA), 150 nM of each primer set, 100 ng of cDNA, along with the master mix was added to each well of a 96-well PCR plate, to a total volume of 10 μL. The qPCR reaction settings are as follows: 95 °C for 5 min followed by a 40-cycle setting at 95 °C for 30 s, 55 °C for 1 min, and 72 °C for 1 min, followed by 95 °C for 30 s, 55 °C for 30 s, and lastly 95 °C for 5 min. A melting curve was added at the end to determine primer specificity for each sample.

### 2.7. Preparation of Samples for RNA-Seq and RNA-Seq Analysis

NHAs and SVGAs were plated to 70% confluence in a 24-well plate. Medium was removed and cells were infected with JCPyV (MOI = 0.1 FFU/cell) in 200 μL or mock-infected with an empty cell lysate preparation control performed in triplicate wells for each treatment. Cells were incubated at 37 °C for 1 h in the respective medium and following infection the cells were fed with 1 mL of media and incubated at 37 °C for 24, 48, and 96 h. At each time point, cells were either fixed and stained for T Ag or 200 μL of TRIzol reagent (Invitrogen, Waltham, MA, USA) was added to each well and stored in the −20 °C. After validating similar levels of infection in NHAs and SVGAs at each timepoint by indirect immunofluorescence assay, cells in TRIzol reagent were removed from the −20 °C storage and total RNA was extracted from each sample with the Direct-zol RNA kits according to the manufacturer’s protocol (Zymo Research, Irvine, CA, USA). RNA quantification was determined by using the NanoDrop One, both mRNA libraries were prepared, and samples were sequenced at Beckman Coulter Genomics (Genewiz, South Plainfield, NJ, USA). Remaining RNA was converted to cDNA and both T Ag and VP1 transcript was measured by qPCR as previously reported [32] to validate transcript levels at each time point.

Strand-specific RNA-seq was prepared, and the paired-end reads were sequenced on an Illumina HiSeq with 20 to 30 million reads per sample with poly(A) selection, using next generation sequencing technology. Quality control was performed on the sequenced reads using FastQC (Version 0.11.8) (http://www.bioinformatics.babraham.ac.uk/projects/fastqc/, accessed on 24 April 2019) and reads were trimmed using Trimmomatic (Version 0.39) [68]. Trimmed reads were then aligned to the human genome assembly (Version GRCh38) using HISAT2 [69] and read counts per gene were generated using the Ensembl GTF file (Ensembl version 103) and HTSeq (Version 0.13.5) [70]. The read counts per gene were analyzed using RStudio (version 1.2.1335) and R/edgeR (version 3.30.3) [71].

### 2.8. STRING Interaction Database, GO Enrichment Analysis, and PANTHER Pathway Analysis

Interaction of genes, including genes in the MAPK/ERK cascade, were visualized using STRING (http://string-db.org, accessed on 28 May 2019). STRING is a database that illustrates the direct and indirect, or the physical and functional association, respectively, of two or more proteins [72]. Interactions of genes were defined as indicated in the figure legends. Gene networks were imported into Cytoscape (Version 3.8.2) to merge information from the R/EdgeR analysis. Genes were also selected based on the EGF-EGFR-RAS-ERK signaling pathway (ID: N00001) from the Kyoto Encyclopedia of Genes and Genomes (KEGG) database [73,74,75] by obtaining the gene symbols and then converting them to Ensembl Gene IDs using BioMart [76]. Lastly, the list of genes from the STRING database were used for a Gene Ontology enrichment analysis using PANTHER [77]. The PANTHER pathway terms corresponding to the gene list were visualized using the R package, GOplot [78].

### 2.9. Statistical Analysis and Graphing in RStudio

For data that was normally distributed, the two-sample Student’s *t* test assuming unequal variances was used to compare the mean values for at least triplicate samples. However, for data that was not normally distributed, the Wilcoxon signed-rank test was used to compare the median values for two populations. For comparing more than two samples the one-way analysis of variance (ANOVA), or the Kruskal–Wallis test, was used depending on the distribution of the data. If the Kruskal–Wallis test determined a significant difference between groups, the pairwise Wilcoxon rank-sum test, along with the Bonferroni adjustment, was used to determine the pair of groups that were different. Distribution of the data was determined by the Shapiro–Wilk’s test and a quantile–quantile plot (Q–Q plot), a plot to illustrate normality of the data, in RStudio (version 1.2.1335). All statistical analyses were performed in RStudio, other than the Student’s *t*-test, which was determined in Microsoft Excel. All statistical analyses referring to the RNA-seq data was performed using the R/edgeR (version 3.30.3) [71], including the MA plots. All graphs, including heatmaps were creating using ggplot2 (version 3.3.3) [79].

## 3. Results

### 3.1. RNA-Seq Reveals Unique Differential Gene Expression in JCPyV-Infected Primary Astrocytes

**Figure 1:**
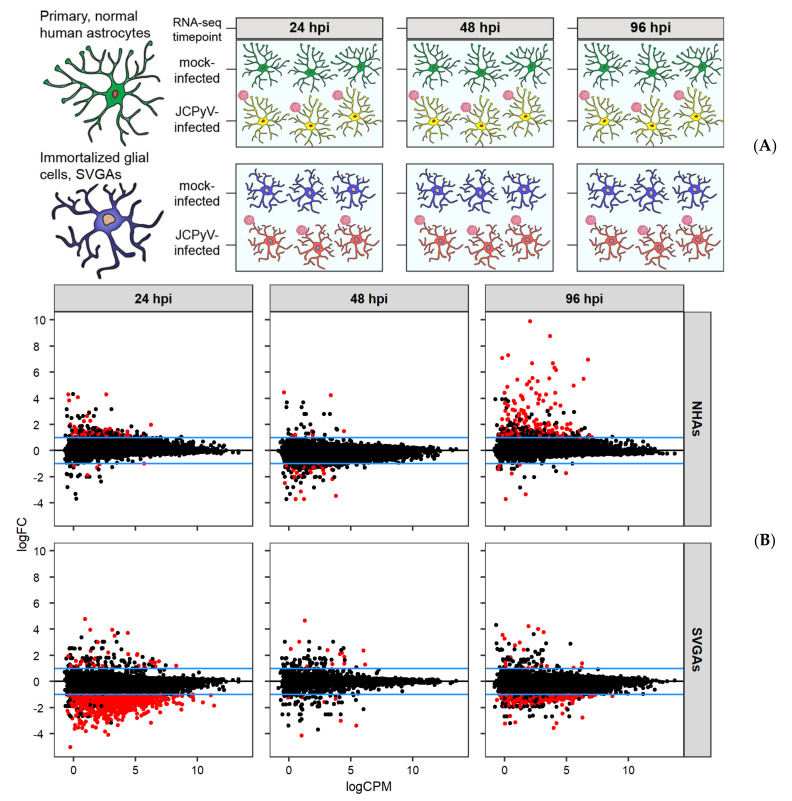
Whole transcriptome RNA-seq profiles of JCPyV infected primary astrocytes and SVGAs during course of infection reveal unique differential gene expression in primary cells. (**A**) Primary, normal human astrocytes (NHAs) and immortalized glial cells (SVGAs) were either infected with JCPyV (MOI = 0.1 FFU/cell) or mock-infected with a vehicle control, and the transcriptomic profile was determined at 24, 48, and 96 hpi. (**B**) RNA-seq data were normalized with the read CPM method and MA plots were generated from DEseq2 analysis at each timepoint representing the log_2_ fold changes of JCPyV-infected NHAs versus mock-infected NHAs, or JCPyV-infected SVGAs versus mock-infected SVGAs. Black points are genes from the R/EdgeR analysis. Red points represent genes with a logFC greater or less than 1 and an unadjusted *p* value < 0.05. The blue lines in each plot represent the logFC = 1 and logFC = −1; FC: Fold-change; CPM: Counts per million.

RNA-seq was performed to measure gene expression in NHAs that were either mock- or JCPyV-infected at 24, 48, and 96 hpi, as illustrated in Figure 1A. SVGAs were used as a reference cell line and to further define cell-type dependent differences that revealed a delay in JCPyV infection in NHAs [32]. Time points selected were previously identified as critically different in both early and late JCPyV transcript and protein production in NHAs compared to SVGAs [32]. Specifically, there was no difference in early gene transcript between NHAs and SVGAs at 24 hpi, a significant increase in late gene transcript was only observed in SVGAs at 48 hpi, and VP1 production was more notably produced in NHAs at 96 hpi [32]. Due to these differences, differential gene expression was determined for NHAs and SVGAs at each time point. To compare the magnitude of genes that were differentially expressed from JCPyV infection in NHAs versus SVGAs, a statistical and expression criterion was established, represented as red points in Figure 1B (unadjusted *p* < 0.05 and log_2_FC > 1 or log_2_FC < −1). At 24 hpi, 95 genes were upregulated and 21 genes were downregulated in NHAs, versus only 51 genes upregulated and 1123 genes downregulated in SVGAs (Figure 1B). At 48 hpi, the relationships between genes having a log fold change above or below one with an unadjusted *p* value less than 0.05 were reversed for each cell type: in NHAs, 28 genes were activated and 72 genes were repressed, and in SVGAs, 25 genes were upregulated and 17 genes downregulated. Finally, the 96 hpi time point mirrored the relationship found at 24 hpi for both cell types. In NHAs, more genes were upregulated (301 genes) and only 33 genes were downregulated, versus SVGAs where 286 genes were downregulated and only 34 genes were upregulated at 96 hpi (Figure 1B). Taken together, these data reveal a pattern of differential gene expression over the course of JCPyV infection that is uniquely different in primary human astrocytes in comparison to an immortalized glial cell line.

### 3.2. The MAPK/ERK Pathway Is Differentially Expressed in NHAs during JCPyV Infection

**Figure 2:**
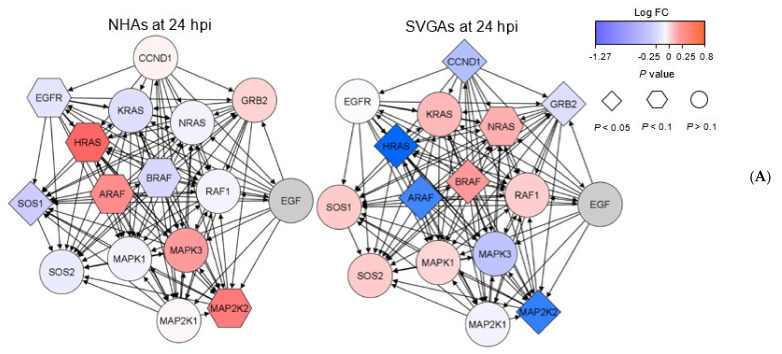

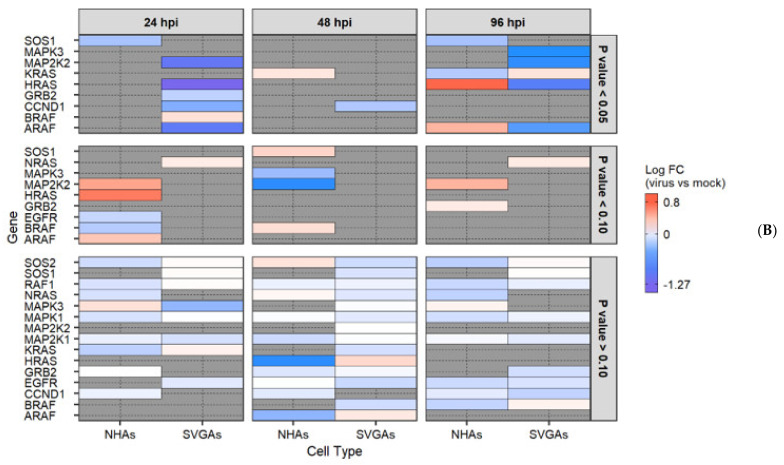
The MAPK/ERK1/2 cascade and associated gene networks are differentially expressed in NHAs compared to SVGAs during JCPyV infection. (**A**) Genes of the MAPK-ERK1/2 were entered into the STRING interaction database to determine the protein–protein interactions determined experimentally or from curated databases of the immediate, surrounding genes within the MAPK/ERK1/2 cascade of NHAs (left) or SVGAs (right). These interactions were defined as having an interaction score of 0.9 with no more than 10 interactors in the 1st and 2nd shell. Each node (i.e., gene) was colored based on the log fold change of JCPyV-infected cells to mock-infected cells at 24 h, and the shape of the gene represents the significance. Arrows point from the source gene to the target gene. Genes colored gray were in the network but were filtered out of the RNA-seq analysis. (**B**) Heat map indicates the Log_2_ fold change of JCPyV-infected cells versus mock-infected cells of the genes within the MAPK/ERK1/2 cascade faceted by timepoint (24, 48, and 96 hpi) and *p* value (*p* < 0.05, *p* < 0.10, and *p* > 0.10).

The MAPK/ERK pathway has been previously established to regulate JCPyV infection in both SVGAs and primary RPTECs [40,41,43]. This regulatory network is influenced by numerous genes upstream and downstream of the pathway. To further elucidate the differential gene expression observed in Figure 1B, genes within the network of the MAPK/ERK pathway were analyzed (Figure 2). These genes were determined using the KEGG database [73,74,75], in the EGF-EGFR-RAS-ERK signaling pathway (ID: N00001). The interactions of these genes were determined using the STRING interaction database (Figure 2A) and examined in the RNA-seq data to determine the cell-specific expression patterns, examining both the log fold change and unadjusted *p* value for each gene in NHAs and SVGAs at 24 hpi (Figure 2B). In NHAs, there were eight genes that had a negative log fold change, and *SOS1* (SOS1) had an unadjusted *p* value threshold of less than 0.05 (Figure 2A, left). In the MAPK/ERK cascade specifically, *HRAS* (HRAS)*, MAP2K2* (MEK2), and *MAPK3* (ERK1) had a positive log fold change, however only *HRAS* (HRAS) and *MAP2K2* (MEK2) had an unadjusted *p* value less than 0.1. This contrasted with SVGAs, where *HRAS* (HRAS)*, MAP2K2* (MEK2)*,* and *MAPK3* (ERK1) had a negative fold change and a *p* value less than 0.05 for *HRAS* (HRAS) and *MAP2K2* (MEK2) (Figure 2A, right). To understand how the MAPK/ERK cascade changes over the course of JCPyV infection, the log fold change of these genes for each time point in both NHAs and SVGAs was determined and illustrated as a heatmap in Figure 2B, faceted by *p* value to further reveal significance. The differing gene expression observed at 24 hpi between NHAs versus SVGAs was also apparent at 48 and 96 hpi. This pattern was most pronounced with the following genes: *HRAS* (HRAS)*, MAP2K2* (MEK2), and *MAPK3* (ERK1). At 48 hpi, many genes were not differentially expressed in SVGAs (Figure 1B and Figure 2B), which is most likely attributed to the JCPyV infectious cycle switching from early to late gene production [32]. However, the expression of genes during JCPyV infection in NHAs, most notably of *MAP2K2* (MEK2) demonstrated a significant difference in fold change from the previous 24-h time point (Figure 2B). Collectively, these data illustrate that the temporal patterns of the genes in the MAPK/ERK pathway during JCPyV infection in NHAs is distinct from and contrary to the MAPK/ERK gene expression pattern in SVGAs, and this distinction can be expanded to the genes surrounding the MAPK/ERK cascade, specifically at 24 hpi.

### 3.3. JCPyV Requires ERK1/2 for Successful Infection in NHAs

**Figure 3:**
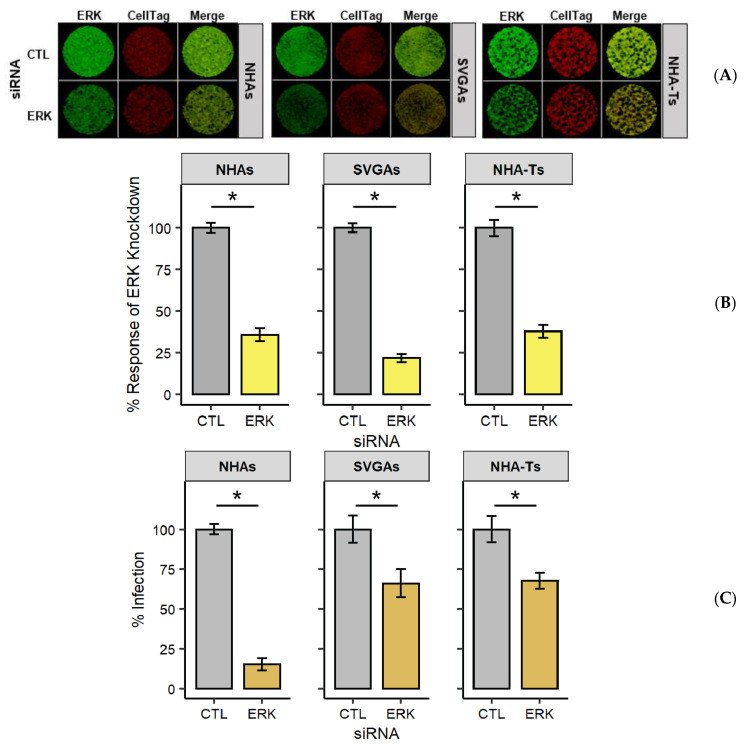
ERK knockdown significantly reduced JCPyV infection in NHAs. NHAs, SVGAs, and NHA-Ts were transfected with ERK1/2 or EGFR (CTL) siRNA. At 72 h post-transfection, cells were fixed for analysis of ERK protein expression (**A**,**B**) or infected (**C**). (**A**) ERK1/2 protein expression was determined by in-cell western (ICW), staining for Total ERK1/2 (green) or CellTag (red). (**B**) Percentage of protein knockdown was measured by ICW signal intensity values per [(ERK1/2)/Cell Tag × 100% = % response] within each ICW analysis using LI-COR software. Error bars indicate SD. Student’s *t*-test was used to determine statistical significance comparing EGFR (CTL) siRNA-treated cells to ERK1/2 siRNA-treated cells, for each cell type. *, *p* < 0.01. (**C**) Following 3 days post-transfection, cells were infected with JCPyV (MOI = 1.0 FFU/cell) at 37 °C for 1 h. Cells were incubated in complete media at 37 °C for 48 h and then fixed and stained by indirect immunofluorescence. Infectivity following ERK1/2 knockdown (**C**) was determined by counting the number of JCPyV T Ag-positive nuclei divided by the number of DAPI-positive nuclei for five × 20 fields of view for triplicate samples. Error bars indicate SD. Student’s *t* test was used to determine statistical significance comparing EGFR (CTL) siRNA-treated cells to ERK1/2 siRNA-treated cells for each cell type (**C**). * *p* < 0.01.

Many genes within the MAPK/ERK cascade had a *p* value > 0.10, and thus, it was of interest to further characterize whether there were differences in the utilization of the MAPK/ERK cascade in NHAs in comparison to SVGAs [40,41,43]. To accurately compare the differences observed in the MAPK/ERK pathway in NHAs and SVGAs, NHA-Ts, which are NHAs immortalized with SV40 T Ag, were used in parallel with the other two cell types [32]. ERK1/2, the final kinase in the MAPK/ERK cascade, was targeted with siRNA in all cell types and subsequently infected with JCPyV at 72 h post siRNA transfection. Protein knockdown of ERK1/2 was analyzed by in-cell western (ICW) (Figure 3A) and quantified using LI-COR software (Figure 3B), a technique extensively validated as a reliable method to quantitate protein expression [43,63,80,81]. When compared to the EGFR control siRNA, there was a ~70% reduction in ERK1/2 achieved in all cell types at 72 h post-transfection (Figure 3B). Following siRNA treatment, all cell types were infected with JCPyV, and infectivity was measured at 48 hpi for cells treated with the ERK1/2 siRNA (Figure 3C). Knockdown of ERK1/2 resulted in a decrease in JCPyV infection in all cell types, although a more appreciable decrease was observed in NHAs, reducing JCPyV infection by ~80% (Figure 3C). These experiments demonstrated the necessity of ERK1/2 during JCPyV infection in NHAs. When similar levels of knockdown were achieved, JCPyV infection was more sensitive to ERK1/2 reduction in primary cells compared to cells transformed with SV40 T Ag.

### 3.4. DUSP1 Transcript Decreases during JCPyV Infection in NHAs and Is Essential in Regulating the MAPK/ERK Pathway Compared to Immortalized Cells

**Figure 4:**
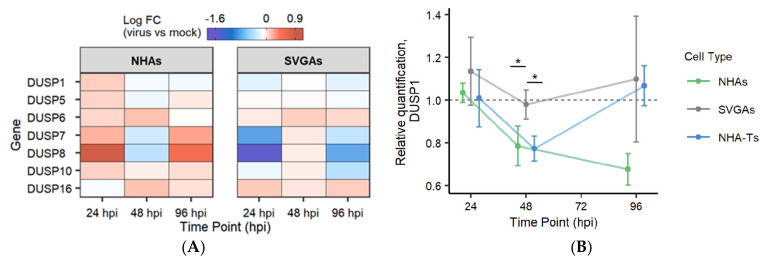
DUSPs were differentially expressed early during infection. (**A**) Heat map indicates the log fold change of typical DUSPs differentially expressed in NHAs and SVGAs at 24, 48, and 96 hpi, derived from the RNA-Seq data. (**B**) NHAs, SVGAs, and NHA-Ts were infected with JCPyV (MOI = 0.1 FFU/cell) or the mock-infected vehicle control at 37 °C for 1 h and incubated in complete media for 0, 24, 48, and 96 h. At each time point, RNA was extracted, and host transcript levels were determined by qPCR. Data represent the relative quantification of DUSP1 transcript in infected cells compared to uninfected cells, first normalized to the Ct values of GAPDH for each treatment (2^−ΔΔ*CT*^). Error bars indicate SD. Data are representative of the average fold change calculated from triplicate samples from three independent experiments. One-way analysis of variance (ANOVA) was used to determine statistical significance comparing the relative quantification of DUSP1 in NHAs, NHA-Ts, and SVGAs at each timepoint. *, *p* < 0.05.

DUSPs are key regulators in the MAPK/ERK pathway, important for the magnitude, duration, and spatiotemporal expression patterns of MAPKs [48,49,50]. Due to the spatial and temporal regulation in differential gene expression (Figure 1B), specifically within the MAPK/ERK pathway (Figure 2B), and the implication of DUSPs in viral infection, the role of these phosphatases was investigated to determine whether they play a role in JCPyV infection and regulation of the MAPK/ERK pathway in NHAs. RNA-seq data revealed that the majority of typical DUSPs (i.e., DUSPs that have either the MAP kinase-binding (MKB) motif or the kinase-interacting motif (KIM)) [51] were upregulated at 24 hpi in NHAs compared to SVGAs, including DUSP1, DUSP6, DUSP7, and DUSP8 (Figure 4A). However, DUSP1 demonstrated an interesting pattern of reduction in NHAs at 48 hpi and remained lower at 96 hpi, as JCPyV infection progressed (Figure 4A). To validate these findings, both DUSP1 transcript levels and expression were further evaluated. During the progression of JCPyV infection, DUSP1 mRNA transcript levels significantly decreased in NHAs compared to SVGAs at 48 hpi, and this reduction continued at 96 hpi, corresponding to trends observed in the RNA-seq data (Figure 4A,B). Altogether, this data illuminates a potential role for DUSPs, specifically DUSP1, during the JCPyV infectious cycle in NHAs.

### 3.5. DUSP1 Is Required for JCPyV Infection in NHAs

**Figure 5:**
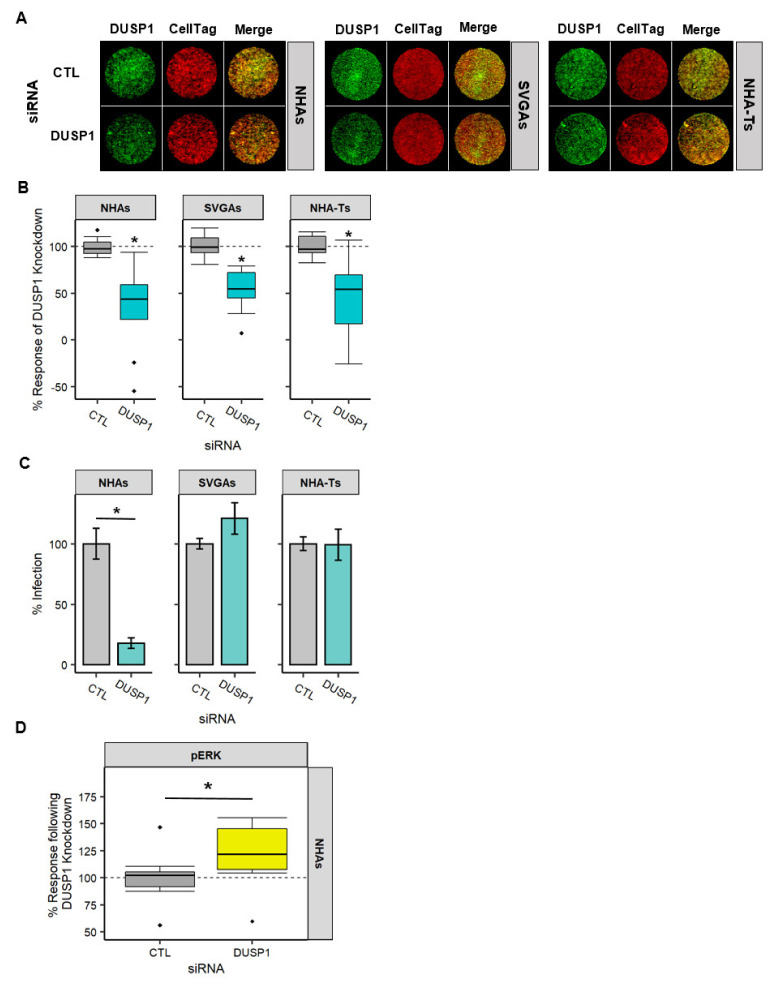
DUSP1 was required for JCPyV infection in NHAs, regardless of pERK expression. NHAs, SVGAs, and NHA-Ts were transfected with DUSP1 or EGFR (CTL) siRNA. At 24 h post-transfection, cells were fixed, imaged, and quantified for protein knockdown (**A**,**B**) or infected (**C**). (**A**) DUSP1 protein expression was determined by ICW, staining for total DUSP1 (green) or CellTag (red). (**B**) Quantification of DUSP1 protein expression was measured by ICW signal intensity values per [(DUSP1)/Cell Tag × 100% = % response] within each ICW analysis in LI-COR software. Cells treated with DUSP1 siRNA were normalized to the respective EGFR (CTL) siRNA (dashed line). Box and whisker plots represent the distribution of 9 samples, with the median denoted by the black line and whiskers representing values 1.0 times the distance of the inter-quartile range. Outliers are represented by black diamonds. A pairwise Wilcoxon rank-sum test, along with the Bonferroni adjustment, was used to compare CTL and DUSP1 siRNA-treated cells across each cell type. *, *p* < 0.01. Data are representative of three independent experiments performed in triplicate. (**C**) At 24 h post-transfection, cells were infected with JCPyV (MOI = 1.0 FFU/cell) at 37 °C for 1 h. Cells were incubated in complete media for 48 h then fixed and stained by indirect immunofluorescence. Percent infection following DUSP1 knockdown was determined by quantifying the number of JCPyV T Ag-positive nuclei divided by the number of DAPI-positive nuclei for five × 20 fields of view for triplicate samples. Error bars indicate SD. Student’s *t* test was used to determine statistical significance comparing EGFR (CTL) siRNA-treated cells to DUSP1 siRNA-treated cells, for each cell type. *, *p* < 0.01. (**D**) Quantification of pERK expression following DUSP1 siRNA at 24 h post-transfection was measured by ICW signal intensity values per [(pERK)/Cell Tag × 100% = % response] within each ICW analysis in LI-COR software. Cells treated with DUSP1 siRNA were normalized to the EGFR (CTL) siRNA (dashed line). Box and whisker plots represent the distribution of 9 samples, with the median denoted by the black line and whiskers representing values 1.0 times the distance of the inter-quartile range. Outliers are represented by black diamonds. A Wilcoxon rank-sum exact test was used to compare CTL and DUSP1 siRNA-treated cells. *, *p* < 0.05.

RNA-seq revealed noticeable trends of DUSP expression during JCPyV infection in NHAs, especially compared to SVGAs and NHA-Ts (Figure 4). This data, taken together with the temporal gene expression observed in the MAPK/ERK cascade (Figure 2), and DUSP1 involvement in other viral infections [56,57,58,59], suggested that DUSP1 may play a role in JCPyV infection. DUSP1 was targeted by siRNA knockdown in NHAs, SVGAs, and NHA-Ts to probe whether it influenced JCPyV infection (Figure 5). Protein knockdown was analyzed by ICW (Figure 5A) and quantified using LI-COR software [43,63,80,81] (Figure 5B). Reduction of DUSP1 protein levels was significantly lower compared to the control siRNA in all cell types at 24 h post siRNA treatment (Figure 5B). Following protein knockdown, all cell types were infected with JCPyV, and infection was scored at 48 hpi by indirect immunofluorescence (Figure 5C). DUSP1 knockdown significantly reduced JCPyV infection in NHAs yet had no impact on infection in SVGAs or NHA-Ts (Figure 5C). These findings were surprising as DUSP1 is a negative regulator of ERK [50], thus ERK1/2 phosphorylation following DUSP1 siRNA in NHAs was also measured (Figure 5D). Following DUSP1 siRNA treatment, pERK expression was significantly increased in NHAs (Figure 5D). Collectively, this data demonstrates that DUSP1 expression is required for JCPyV infection in NHAs, yet surprisingly the knockdown of DUSP1 in NHAs did not negatively impede the MAPK/ERK cascade.

### 3.6. The Network of Genes Related to the Interactions of DUSP1 and ERK1/2 Are Involved in the Pathways of the Immune Response, Cell Survival, and Apoptosis

**Figure 6:**
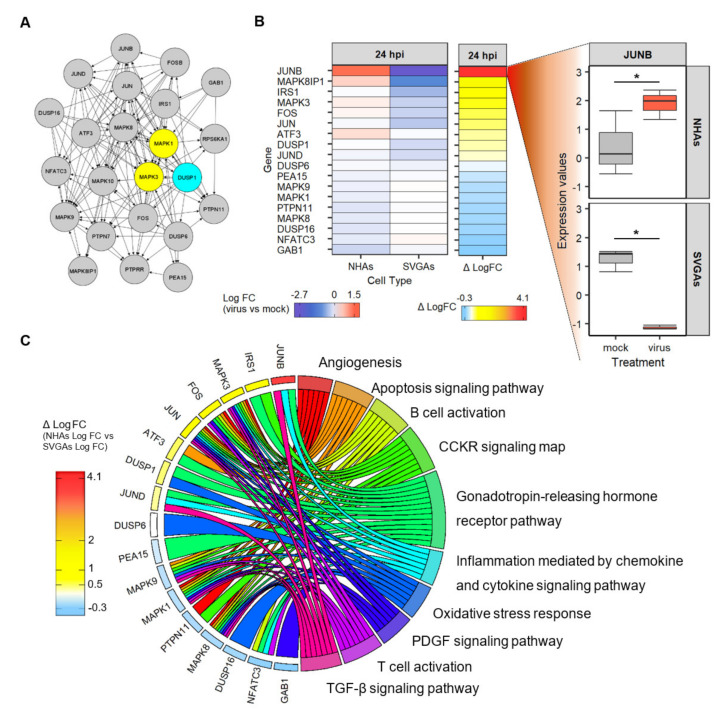
PANTHER pathway analysis of the genes within the DUSP1–ERK1/2 network, specifically JUNB, reveals pathways in immune activation and downstream activation of the receptor tyrosine kinase pathways. (**A**) Genes ERK1/2 and DUSP1 were analyzed in the STRING interaction database and the network was expanded three times to determine the protein–protein interactions of both known and predicted interactions of the surrounding genes. These interactions were defined as having an interaction score of 0.4 with no more than 10 interactors in the 1st and 2nd shell. Arrows point from the source gene to the target gene. (**B**) The heat map illustrates the log_2_ fold change of JCPyV-infected versus mock-infected NHAs and SVGAs for all genes and the Δ log_2_ fold change between cell types from the previous network that were differentially expressed at 24 h. The expression values (log CPM) of JUNB were determined, illustrated by box and whisker plots with the distribution of 3 samples, with the median denoted by the black line and whiskers representing values 1.5 times the distance of the inter-quartile range. *, *p* < 0.05. (**C**) PANTHER pathway analysis was used to identify the pathways from the genes in the ERK1/2 and DUSP1 network. A GOChord plot represents the top ten pathways, representing at least 35% of the 17 genes in the network. Each gene is linked via ribbons to their assigned PANTHER pathway term and the colored bars next to each gene represent the difference between the log_2_ fold change in JCPyV-infected versus mock-infected NHAs and between JCPyV-infected versus mock-infected SVGAs. The gene, *MAPK8IP1* was not included in the PANTHER pathway analysis as it is not in the PANTHER pathway database.

Results from DUSP1 siRNA (Figure 5) elucidated other mechanisms or pathways JCPyV may modulate during infection of NHAs, not observed in SVGAs and NHA-Ts. To understand the cellular pathways and the downstream impacts that may be involved, the genes DUSP1 (DUSP1), MAPK3 (ERK1), and MAPK1 (ERK2) were entered into the STRING interaction database and an over-representation analysis was performed using the PANTHER (protein analysis through evolutionary relationships) pathway database at 24 hpi (Figure 6). The network was expanded three times to elucidate the genes in the immediate proximity of DUSP1 (DUSP1), MAPK3 (ERK1), and MAPK1 (ERK2) having known and predicted interactions with other genes and a medium confidence interaction score, with no more than 10 interactors in the first and second shell (Figure 6A). These genes were then analyzed in the RNA-seq data to determine the log fold change in NHAs and SVGAs at 24 h, and are summarized as a heatmap (Figure 6B). These data illustrate that largely, genes within the DUSP1 and ERK1/2 network have a negative fold change in SVGAs during JCPyV infection; however, MAPK3 (ERK1), ATF3 (ATF3), JUNB (Jun-B), and MAPK8IP1 (c-Jun-amino-terminal kinase-interacting protein 1) were differentially expressed in NHAs during viral infection (Figure 6B). In addition, this analysis revealed a significant fold change increase in JUNB (Jun-B) during JCPyV infection in NHAs, in comparison to a significant fold change decrease observed in SVGAs (Figure 6B). To further understand the biological function of the network of genes from the interactions of DUSP1 and ERK1/2, the list was entered into the Gene Ontology website and an over-representation analysis was performed, using the PANTHER pathway database (Figure 6C). The GoChord plot summarizes the 17 genes, which are joined with ribbons to their respective cellular pathways. (MAPK8IP1 was not included as it is not in the PANTHER database.) The legend defines the difference between the log fold change of NHAs and the log fold change of SVGAs to simplify the output. Genes that are orange/yellow had a large positive difference between NHAs and SVGAs, and genes colored blue had a small or negative change between cell types during JCPyV infection. Genes that had a greater positive change (i.e., JUNB [Jun-B], IRS1 [IRS1], MAPK3 (ERK1), FOS [c-Fos], JUN [Jun], and ATF3 [ATF3]) were associated with pathways of inflammation mediated by chemokine and cytokine signaling pathways, including TGF-β signaling, apoptotic, and cholecystokinin receptors (CCKR) signaling pathways (Figure 6C). These data suggest that in primary astrocytes, the network of genes associated with DUSP1 (DUSP1), MAPK3 (ERK1), and MAPK1 (ERK2) are important during JCPyV infection for both the host and the virus. Primary astrocytes may induce an immune response by releasing cytokines and chemokines early during infection, not seen in SVGAs, but in turn, the virus slowly activates transcription factors, like Smad4 from the TGF-β signaling pathway to support viral replication in the nucleus of NHAs [43].

## 4. Discussion

**Figure 7:**
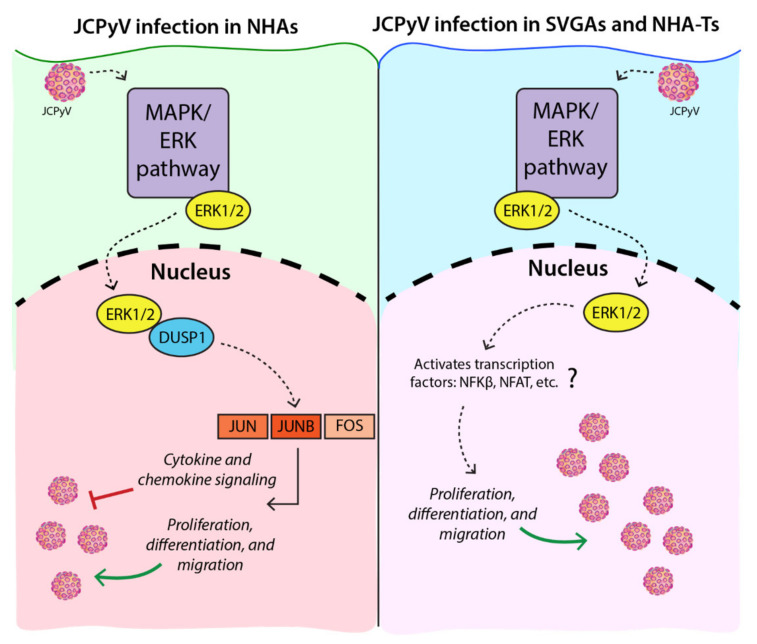
The MAPK/ERK pathway is required for JCPyV infection in primary and immortalized astrocytes but may activate additional pathways in primary astrocytes, specifically pathways involved in the immune response and facilitated by the transcription factor, *JUNB*, and other members to form the AP-1 complex. This may result in the inhibition and the delay of JCPyV infection observed in NHAs compared to immortalized astrocytes [32].

JCPyV specifically targets glial cells in the CNS, including astrocytes, which have been implicated as a potential reservoir for infection, eventually leading to the pathogenesis of PML [5]. However, due to limited understanding of gene regulation in JCPyV infection, our knowledge of JCPyV progression in astrocytes is incomplete. Thus, RNA-seq analysis was performed using primary human astrocytes infected with JCPyV at time points previously established to demonstrate marked differences in the production of viral T Ag and VP1 in NHAs and SVGAs (Figure 1A) [32]. RNA-seq revealed a significant, distinct transcriptomic profile in NHAs during JCPyV infection when compared to the more commonly studied immortalized glial cell line, SVGAs. This was illustrated by the temporal difference in gene expression of the MAPK/ERK pathway during infection and illuminated a critical role for DUSPs, specifically DUSP1, during JCPyV infection in NHAs. The role of ERK1/2 and DUSP1 were further validated using protein expression assays and protein inhibition by siRNA, revealing the notable role these proteins have during JCPyV infection in NHAs (Figure 7). Utilizing additional bioinformatics techniques, we elucidated novel pathways activated during JCPyV infection in NHAs.

Our laboratory recently determined that the JCPyV infectious cycle in NHAs was delayed compared to the infectious cycle in SVGAs [32]. RNA-seq analysis revealed that at 24 hpi, a significant number of genes that were differentially expressed in SVGAs were downregulated during JCPyV infection; comparatively, more genes were upregulated in NHAs (Figure 1B). At 48 hpi, most genes differentially expressed in NHAs were downregulated, resembling the 24-h time point in SVGAs (Figure 1B), whereas ~75% of genes (*p* < 0.05) that were downregulated in NHAs at 48 hpi were downregulated in SVGAs at 24 hpi (data not shown). These data suggest that the delay of JCPyV infection in NHAs, as previously described, may be regulated in part by the virus establishing infection more efficiently in SVGAs as numerous genes may already be downregulated due to the immortalized characteristics of the cell type [36,37]; however, SVGAs are also a heterogeneous population of cells [29].

To further define astrocytic infection, the MAPK/ERK pathway was explored, as it has been demonstrated to be a critical regulator of JCPyV infection in SVGAs and other cell types [40,41]. The MAPK/ERK pathway activates various genes and transcription factors through a cascade of kinases that orchestrate events for cellular proliferation, differentiation, and survival [42]. Given this evidence, the MAPK/ERK pathway and genes with protein–protein interactions were examined in the RNA-seq data set (Figure 2). Several kinases within the pathway were differentially expressed, most notably *HRAS* (HRAS) and *MAP2K2* (MEK2) at 24 and 96 hpi in NHAs (Figure 2B). Expanding this analysis to genes that interact with the MAPK/ERK pathway also revealed differentially expressed genes. For example, at 24 hpi, *SOS1* (SOS1) was significantly downregulated in NHAs. *SOS1* (SOS1) is a critical linker between tyrosine kinase receptors and an activator of *KRAS* (KRAS) in the MAPK/ERK pathway, as it interacts with *KRAS* (KRAS) to potentially influence the duration and magnitude of ERK activation [82,83,84]. Genes determined to have protein–protein interactions, such as *SOS1* (SOS1), in the MAPK/ERK pathway were downregulated at 24 hpi (Figure 2A). RNA-seq results also revealed temporal, differential gene expression over the course of infection in both cell types from 24 to 96 hpi.

It has been previously reported that MEK1/2 and ERK1/2 phosphorylation are biphasic during JCPyV infection [40,43], and these data substantiate the biphasic expression of not only ERK1/2 but the other kinases in the pathway. Interestingly, the gene expression of these kinases was temporally regulated in NHAs and SVGAs, yet their expression was non-concurrent at each timepoint (Figure 2). These data establish a preliminary understanding of the MAPK/ERK pathway in NHAs; however, they reveal differential expression and activation dynamics between cell types for MAPK/ERK pathway utilization during viral infection. To validate the RNA-seq results and further define the importance of the MAPK/ERK pathway in NHAs during JCPyV infection, ERK1/2 were targeted for knockdown using siRNA (Figure 3). Knocking down ERK1/2 resulted in a significant decrease in JCPyV infection in NHAs, confirming that the requirement for ERK1/2 in JCPyV infection extends to primary astrocytes [40]. Surprisingly however, DUSP1, a key negative regulatory protein of the MAPK/ERK pathway [47,48,49,50] did not increase JCPyV infection upon DUSP1 siRNA knockdown. In addition, the expression of DUSP1 was downregulated at 48 and 96 hpi (Figure 4A), as well as DUSP1 transcript at 48 hpi, compared to JCPyV infection in SVGAs (Figure 4B). Although these changes were minimal, taken together, this data suggests that as viral infection ensues in NHAs and the MAPK/ERK pathway is activated, the negative regulatory role of DUSP1 is hindered, which ultimately hampers the activation of alternative pathways that could disrupt viral replication. However, because the MAPK/ERK pathway is activated early during JCPyV infection [40,41,43], future RNA-seq studies should examine earlier timepoints to validate these results and define how cellular pathways can trigger certain transcription factors to support JCPyV infection.

DUSP1 siRNA treatment resulted in a significant decrease in JCPyV infection in NHAs, but there was no difference during infection of immortalized cells (Figure 5). This indicates that DUSP1 is dispensable during JCPyV infection in cells immortalized with SV40 T Ag, as reduction of this phosphatase did not alter viral infection. This was also observed in RSV infection of A549 cells, in which silencing DUSP1 did not alter viral replication [58]. However, previous research has demonstrated that cells transformed with oncogenes can influence the expression of protein phosphatase 2A (PP2A) [85,86,87]. PP2A has a similar role to DUSP1 by acting as a negative regulator of the MAPK/ERK pathway [88]. Therefore, in transformed cells it is possible that expression of small T antigen antagonizes PP2A, in which case DUSP1 would not be required. Further research should elucidate the role of PP2A during DUSP1 knockdown and JCPyV infection to determine if PP2A is compensating for the loss of DUSP1 expression in transformed cells. Conversely, the decrease in infection observed in NHAs is comparable to the results found by Choi et al. [57]. HCV infection was significantly reduced upon silencing DUSP1, in which authors demonstrated that this was due to STAT1 translocation to the nucleus, increasing expression of ISGs and thus suppressing HCV infection [57]. Similarly, Assetta et al. demonstrated that pSTAT1 was localized to nuclei in JCPyV-infected cells, leading to the activation of ISGs, and this contributed to the persistent nature of JCPyV infection in a primary kidney cell model [39]. These previous findings provide the premise to hypothesize that when DUSP1 is knocked down during JCPyV infection in NHAs, viral infection is reduced because STAT1 is translocated to the nucleus, activating ISGs and thus inhibiting JCPyV infection. Even when ERK1/2 is phosphorylated upon DUSP1 reduction (Figure 5D), the host response to viral infection may overcome the additional activation of ERK1/2. However, more research is required to test this hypothesis. Additionally, taken together with the previous results (Figure 4), RNA-seq analysis determined that DUSP1 expression is important early in the process of JCPyV infection (Figure 4A), and this may play a role in suppressing the immune response during early events of the infectious cycle in NHAs, whereas reducing DUSP1 through siRNA significantly decreased JCPyV infection (Figure 5C).

Research has demonstrated that DUSP1 has a critical role in the inflammatory response against various pathogens [57,60,89,90,91] and plays an intricate role in regulating pro- and anti-inflammatory cytokines [60]. Furthermore, astrocytes are important in maintaining many neuronal functions in the CNS through production of cytokines and chemokines [92,93]. This ultimately results in astrocytes having a neurotoxic or neuroprotective role in the brain of human neurological diseases [94]. Due to the role of DUSP1 in the inflammatory response and the interaction of ERK1 and ERK2, an over-representation analysis using PANTHER [77] was performed on the list of genes that have known and predicted interactions with these proteins (Figure 6). *JUNB* (Jun-B)*,* has both predicted and known interactions with *DUSP1* (DUSP1)*, MAPK3,* (ERK1), and *MAPK1* (ERK2), determined by the STRING interaction database [72], and was significantly upregulated at 24 hpi in NHAs and significantly downregulated in SVGAs (Figure 6B). Through the PANTHER analysis, *JUNB* (Jun-B), *MAPK3* (ERK1), *JUN* (Jun)*,* and *JUND* (Jun-D) were associated with the inflammation mediated by chemokine and cytokine signaling pathway (PANTHER Accession: P00031) (Figure 6C). Furthermore, as determined by a gene set enrichment analysis, preliminary data suggests that the interleukin 10 (IL-10) signaling pathway is expressed in NHAs at 24 hpi (data not shown). Previous microarray analysis has also considered the involvement of inflammatory responses during JCPyV infection in primary astrocytes as well [38]. This data further establishes that claim, as early during JCPyV infection in NHAs, astrocytes may modulate an immune response that is not activated in SVGAs and may contribute to the delay of JCPyV late protein production observed in NHAs [32].

The TGF-β signaling pathway (PANTHER Accession: P00052) was also associated with genes that had a greater difference in differential gene expression in NHAs compared to SVGAs (Figure 6C). SMAD4 is involved in the TGF-β signaling pathway, regulating transcription factors of target genes in the nucleus [95], and has also been implicated in JCPyV infection [43,96]. Previous research has demonstrated that JCPyV-infected cells expressed higher levels of SMAD4 in the nucleus compared to uninfected cells [43]. Furthermore, the gene *ATF3* (activation transcription factor-3), demonstrated higher expression in infected NHAs compared to SVGAs, and is known to interact with SMAD4 [97]. These findings indicate that early during JCPyV infection, genes associated with *DUSP1* (DUSP1)*, MAPK3* (ERK1), and *MAPK1* (ERK2) are involved in activating pathways that both induce a host response and simultaneously create a conducive environment for viral replication. This evidence provides a foundation for future studies to define how JCPyV infection activates alternative pathways in primary cells not observed in immortalized cells and may provide insight into the delayed infection in NHAs [32] compared to infection in SVGAs.

This research further characterizes JCPyV infection in primary human astrocytes and determines the transcriptomic profile compared to that in SVGAs. These differences were also observed in the timing of MAPK/ERK pathway expression and targeting ERK1/2 via siRNA, validating the importance of this signaling pathway during astrocytic infection. Furthermore, this research highlights a role for DUSPs, specifically DUSP1, during JCPyV infection in NHAs, which is not observed in immortalized cells. Lastly, by analyzing pathways and gene networks we further defined how the interactions of DUSP1, ERK1, and ERK2 may activate novel cellular pathways during JCPyV infection in primary human astrocytes (Figure 7). This research validates and establishes novel mechanisms of JCPyV infection in primary human astrocytes, in turn further elucidating how viral infection can lead to a deadly human disease.

## Figures and Tables

**Table 1:** Antibodies used in immunofluorescence and ICW assays.

Protein	1⁰ Antibody (Dilution, Manufacturer)	2⁰ Antibody (Dilution, Manufacturer)
JCPyV T Ag	PAB962 (1:5, hybridoma)	anti-mouse Alexa Fluor 594 (1:1000, Thermo Fisher Scientific)
Total ERK (p44/42 MAPK)	4695S (1:500, CST)	anti-rabbit IRDye 800CW (1:10,000, LI-COR)
Phospho-p44/42 MAPK (ERK1/2) (T202/Y204)	9101S (1:750, CST)	
Total DUSP1	sc-373841 (1:75, Santa Cruz Biotechnology)	anti-mouse IRDye 800CW (1:10,000, LI-COR)

CST: cell signaling technology; ICW: in-cell western.

## Data Availability

The RNA-seq data were deposited in the National Center for Biotechnology Information (NCBI) Gene Expression Omnibus (GEO) database, under accession number GSE183322.
